# Educating residents in spine surgery: A study of Entrustable professional activities in neurosurgery and orthopedic surgery

**DOI:** 10.1371/journal.pone.0311444

**Published:** 2024-10-04

**Authors:** Janissardhar Skulsampaopol, Yu Ming, Michael D. Cusimano

**Affiliations:** 1 Department of Surgery, Division of Neurosurgery, University of Toronto, Toronto, Ontario, Canada; 2 Faculty of Medicine, Department of Surgery, Ramathibodi Hospital, Mahidol University, Bangkok, Thailand; National Trauma Research Institute, AUSTRALIA

## Abstract

**Background:**

Surgery for spinal disorders represents some of the commonest surgical procedures performed in many countries worldwide, carried out by neurosurgeons and orthopedic surgeons. Residency training is shifting to competency-based medical education, which requires setting standards for graduating residents and their assessments. However, gaps exist in the literature regarding the parameters used for assessment and the mastery levels expected of graduating residents in the performance of common spinal procedures as defined in Entrustable Professional Activities (EPAs). The objectives of the study were to describe the assessment parameters used for residents, identify the standard of performance expected of graduating residents of EPAs of spinal procedures, and identify factors predicting the expected standard of competent performance of graduating residents.

**Methods:**

The survey was sent to neurosurgery and orthopedic surgery Faculty requesting their recommendations on parameters of assessment and the expected standard competence performance for EPAs related to spinal procedures using our entrustment scale (A-E).

**Results:**

Based on total responses, the recommended number of assessments and assessors for each EPA was 5 and 2, respectively. Regarding each specialty, there was no significant difference in the recommended number of assessments for each EPA. However, neurosurgery Faculty recommended higher number of assessors(n = 3) than orthopedic surgery Faculty(n = 2) for both posterior spinal decompression EPA(PSD) (p = 0.01) and spinal instrumentation EPA(SI) (p = 0.04).

Based on total responses, 83% felt PSD was appropriate to the general practice, 86.8% considered it not too broad, and 62.3% expected entrustment level E as a graduation target. The proportions of these ratings were slightly lower for SI at 58.5%, 71.7% and 56.6%, respectively. Both specialties indicated that the EPAs were not too broad. In contrast, neurosurgery Faculty were more likely to consider these EPAs appropriate for general practice than orthopedic surgery Faculty for both PSD (94.7% vs 53.3%, p = 0.0003) and SI (68.4% vs 33.3%, p = 0.02). Moreover, neurosurgery Faculty had a higher expected standard of performance as a graduation target for both PSD (Level E 76.3% vs 26.7%, p = 0.001) and SI (Level E 65.8% vs 33.3%, p = 0.03) than orthopedic surgery Faculty. Expectations of entrustment level E for PSD was associated with the belief that the current EPA was appropriate for the general practice of their specialty with an odds ratio of 8.35 (p = 0.01, 95%CI 1.53–45.67).

**Conclusions:**

A difference exists in parameters of assessment and expected standard competence performance of spine procedures among spinal surgery specialties. In our opinion, there should be efforts to develop consensus between specialties for the sake of uniform delivery of high-quality care for patients regardless of the specialty of their surgeon. Our results will be particularly valuable to certification bodies in the assessment of spinal milestones. This study has important implications for the design of residency and fellowship education in spinal surgery internationally.

## Introduction

Surgery for spinal disorders represents some of the commonest surgical procedures done in many countries around the world [1–10]. They are performed by neurosurgeons and orthopedic surgeons, and both specialties have seen increasing trends for spinal surgery over the last two decades [1–10]. Spine surgery courses are ubiquitous and the popularity of spinal fellowships continues to grow [11]. Spinal procedures are considered a core competency by most neurosurgery training programs around the world.

Since competency-based medical education (CBME) is a form of mastery education, setting the level of competency expected of practicing specialists is a key task of residency programs and certifying bodies. In Canada, the expected standard of competence performance in spinal procedures expected of graduating residents, irrespective of specialty has not been defined. Since numerous certification bodies have implemented the framework of competency-based medical education [12], it has become mandatory that specialties define the essential knowledge, attitudes and behaviors that describe the core competencies required to safely and effectively perform procedures such as spinal decompressions and fusions. An Entrustable Professional Activity (EPA) is defined as a key ***task*** of a discipline (i.e. specialty or subspecialty) that an individual can be trusted to perform in a given health care context, once sufficient competence has been demonstrated [13]. EPAs are a common approach to CBME around the world [12]. While EPAs have been defined for a wide variety of specialties in different countries, a number of issues require further elaboration and clarification.

Since residents must be evaluated on EPAs for competency-based residency education to work, practical questions such as how many evaluations and how many evaluators are required to certify competent performance before a physician could be entrusted to complete a task independently must be answered. The Royal College of Physicians and Surgeons of Canada (RCPSC), states that for posterior spinal decompression EPA (PSD), neurosurgery residents require two assessments and orthopedic residents, one [14,15]. For spinal instrumentation EPA (SI), neurosurgery residents are expected to get a minimum of eight assessments and orthopedic residents are expected to obtain one assessment [14,15]. The Intercollegiate Surgical Curriculum Programme (ISCP) of the United Kingdom (UK) requires 10 assessments of complex spinal fusion for graduating neurosurgery residents and the requirement for orthopedic residents is 20 cases of nerve decompression which could include carpal tunnel, cubital tunnel, tarsal tunnel, spinal decompression and discectomy [16,17]. With respect to the actual number of assessors who must evaluate residents, currently, most spinal surgery EPAs have not stipulated a required number to determine if a resident has reached the point of competence. The one exception is the SI of neurosurgery in Canada which requires at least two different assessors [14–20].

Another aspect that requires clarification if competency-based education for spinal surgery is to succeed, is to determine the actual level of mastery required to determine whether competence around EPAs exists in residents. The Accreditation Council for Graduate Medical Education (ACGME) in the United States indicates that the observable markers of an individual’s ability, or what is known as a “Milestone,” can vary for different stages of residency. The Milestone for neurosurgery residents at a mid-residency point is to be capable of performing laminectomy for stenosis, posterior cervical foraminotomy, 1–2 level ACDF, and open single level instrumented lumbar decompression (Milestones Level 3). For more complex spinal surgery procedures such as corpectomy or 3–4 level ACDF, posterior cervical laminectomy with lateral mass fixation, these are categorized under Milestones Level 4, which is the graduation goal but not a graduation requirement [20]. On the other hand, the graduation target (Level 4) for orthopedic residents is to have the capability to independently perform spinal exposures and assist in PSD and SI [18,19].

It is incumbent on educators to ensure there is some basis and consistency in the parameters of assessment and the mastery levels expected of graduating residents in the performance of common spinal procedures as defined in EPAs. The research presented here addresses this need in education practice and this gap in the literature by providing benchmarks for assessment in spine surgery for residency training as indicated by those training needs.

The objectives of the study were to: 1) describe the parameters of assessment of EPAs of spinal procedures between neurosurgery and orthopedic surgery; 2) identify the standard of performance expected of graduating residents of neurosurgery and orthopedic surgery; 3) identify factors predicting the standard of performance expected of graduating residents.

## Methods

A questionnaire was developed consisting of questions regarding years of practice, number of staff physicians and residents working in the respondents’ specialty at their hospital, number of residents the respondents supervise per day, and the parameters and standards expected of EPAs. The questionnaire was reviewed and pilot tested by sending it to neurosurgery Faculty and revised again before administration to orthopedic Faculty.

The questionnaire included questions regarding procedures related to PSD and SI included in the EPAs of neurosurgery and orthopedic surgery as we planned to determine the similarities and difference in perspectives of the spinal-related EPAs that are common EPAs among neurosurgery and orthopedic surgery. The details of tasks in PSD and SI referencing to RCPSC were slightly different between neurosurgery and orthopedic surgery. These procedures are:

### Posterior spinal decompression EPAs (PSD)

**Neurosurgery: “Performing posterior cervical or thoracic decompression”** which includes proper patient positioning, level confirmation, and *removal of the lamina* while preserving uninvolved ligaments and respecting the spinal cord. This also includes *wider postero-lateral thoracic decompression* for anterior pathology, which involved *resection of the facet joints and pedicle* [14].

**Orthopedic Surgery: “Performing laminectomy/decompression”** which includes correct level localization and verification, safe opening of ligamentum flavum, removal of compressive elements, and appropriate care of neural element(s) [15].

### Spinal instrumentation EPAs (SI)

**Neurosurgery: “Performing procedures utilizing spinal instrumentation including posterior subaxial, posterior thoraco-lumbar, occipito-cervical and anterior cervical”** which includes instrumentation of the spine at *occipito-cervical*, *anterior*, and *posterior cervical*, *posterior thoracic* and *lumbar levels* as well as *lumbar interbody instrumentation* [14].

**Orthopedic Surgery: “Performing primary posterior instrumented spine fusions”** which includes correct level localization and verification, landmarking for pedicle screw start point, insertion of pedicle screw, completion of rod insertion, preparation of fusion site and bone grafting, and appropriate care of neural element(s) [15].

The questions following each EPA regarding parameters of assessment asked participating Faculty for their recommended numbers of assessments (which represents the number of assessments required of each resident before confirming competence) and the number of different assessors required to confirm competence. In addition, participants were requested to provide their opinion regarding the following characteristics of the EPA: 1) if the EPA is suitable for general practice of their specialty or requires fellowship sub-specialization; 2) if the EPA is too broad; and 3) expected competence performance of the EPA for graduating residents.

Regarding the expected standard competence performance for PSD and SI, participants were asked to select one of the five entrustment scales. The scale ranged from A to E, representing lower to higher competency. Levels A-D consist of the standard levels in the RCPSC and we specifically developed and validated a unique and new fifth level that explored the ability of the resident to competently respond to novel complexities that might present to the practicing surgeon and to be able to still do the procedure competently when complexities arise [21,22].

The breakdown of the scale is as follows: A—allowed to observe only, requiring complete guidance or is not currently permitted to perform the activity; B—requiring constant guidance and instruction when performing the activity; C—requiring occasional guidance or instructional prompting when performing the activity; D—*requiring assistance* or instruction only when responding to contextual demands of the activity to ensure patient safety; E—able to adapt performance or decisions in response to contextual complexities of the activity and perform activity *independently* and safely [21,22].

Finally, Faculty were also asked to provide additional open-ended text comments regarding any aspect of the PSD and SI as it relates to their specialty or residents.

#### Survey

An online questionnaire, developed using Survey Monkey ®, was sent to publicly available email addresses of Canadian Faculty members in neurosurgery and orthopedic surgery. We sent the survey to all neurosurgery and orthopedic surgery Faculty which were not only limited to spine surgeons because we would like to get perspectives from the all practicing neurosurgery and orthopedic Faculty regarding their expectation on competency of general as opposed to subspecialty neurosurgeons and orthopedic surgeons on spine-related EPAs during June 14-Septemtber 14, 2021. The study was approved by the Research Ethics Board of Unity Health—St. Michael’s Hospital and each respondent provided informed consent for participation by clicking the "NEXT" button below the consent page to confirm that they had read the form and decided to consent to participate in the study.

#### Statistical analysis

Only complete responses of questions regarding the PSD and SI were analyzed. Descriptive data regarding participants’ working experience were calculated, along with descriptive data about programs, which included number of physicians, number of residents at respondents’ hospital and number of residents under the respondent’s supervision per day.

Median, interquartile range (IQR) and range of number of assessments and number of assessors recommended by neurosurgery and orthopedic surgery Faculty were calculated. These parameters of assessment were compared among the three specialties by using **Wilcoxon rank-sum test**. The proportion of participants considering the EPA appropriate for the general practice of their specialty, those indicating the EPA was not too broad, and the expected competence performance (our novel entrustment level A-E) were calculated and the difference among neurosurgery and orthopedic surgery Faculty was computed using the **Fisher’s exact test**. The level-of-significance was set to 0.05 for all tests.Univariable logistic regression was conducted to examine whether years in practice, number of physicians and residents at the respondents’ hospital, number of residents under the respondent’s supervision per day, and considering the EPA appropriate for general practice of their specialties were associated with the selection of entrustment level E as the expected competence performance for graduating residents.

Statistically significant variables in the univariable model were analyzed in a multivariable regression model. Unadjusted odds ratios (ORs), adjusted odds ratios (aORs) and 95% confidence intervals (CIs) were calculated.

Latent class analysis (LCA) was done on five variables: 1) belief that the current EPA was appropriate for residency training; 2) belief that the current EPA was not too broad; 3) considering entrustment level E as reflecting attainment of competence; 4) belief that more assessments and 5) more assessors were needed. The average number of assessments and assessors were calculated and rounded to the nearest integer. Participants were coded as perceiving the need for ‘more assessments or assessors’ if they indicated a greater number of assessments or assessors than the average. All quantitative analyses were conducted with R [23]. Finally, all open-ended comments were thematically analyzed and demonstrated to help understand the rationale of Faculty’s responses.

## Results

We received a total of 98 questionnaire responses, of which 53 responses were considered complete responses with fully usable data regarding our objectives (usable response rate of 54%). Complete responses for questionnaire items related to PSD and SI were obtained from 53 Canadian neurosurgery (n = 38) and orthopedic Faculty (n = 15). Demographics of respondents to these questions are presented in **Table 1.** As illustrated in **Table 2**, based on total responses of both specialties analyzed together, the recommended number of assessments is 5 and recommended number of assessors is 2 for both PSD and SI.

**Table 1:** Demographics of participants in each specialty.

		Specialty	
		Neurosurgery	Orthopedic Surgery
**Number of participants**		**38**	**15**
**Years of practice** Number (%)	<5 years	10 (26.3%)	1 (6.7%)
	5–10 years	6 (15.8%)	1 (6.7%)
	11–20 years	14 (36.8%)	3 (20.0%)
	> 20 years	8 (21.1%)	10 (66.7%)
**# Faculty practicing at participant’s hospital (median [range])**		9.5 [3–32]	9 [4–24]
**# Residents on participant’s specialty service at participant’s hospital (median [range])**		5 [1–32]	3 [0–10]
**# Residents supervised by participant per day (median [range])**		1.5 [1–5]	1 [0–3]

**Table 2:** Comparison between Faculty perspectives and currently recommended number of assessments and number of assessors for demonstrating resident competence for EPA regarding posterior spinal decompression and spinal instrumentation for each specialty.

EPA	Specialty	Faculty’s recommended number of assessments				Currently required number of assessments by RCPSC	Faculty’s recommended number of assessors				Currently required number of assessors by RCPSC
		Median	IQR	Min	Max		Median	IQR	Min	Max	
Posterior spinal decompression	Neurosurgery	5	7	2	25	2	3	1	1	10	No requirement
	Orthopedic surgery	4	5.5	1	20	1	2	1.5	1	5	No requirement
	P-value	0.18					**0.01** *				
**Overall Study**		5	7	1	25		2	1	1	10	
Spinal instrumentation	Neurosurgery	5	7	3	40	8	3	1	1	10	2
	Orthopedic surgery	4	6	1	25	1	2	2	1	5	No requirement
	P-value	0.17					**0.04** *				
**Overall Study**		5	7	1	40		2	1	1	10	

EPA = Entrustable Professional Activity; RCPSC = The Royal College of Physicians and Surgeons of Canada.

*Statistically significant.

### Posterior spinal decompression EPAs (PSD)

The results pertaining to the PSD are presented in **Tables 2 and 3.** Neurosurgery Faculty suggested a median of 5 assessment while orthopedic surgery Faculty recommended a median of 4 (p = 0.18). The median number of assessors recommended by neurosurgery Faculty was 3, while orthopedic Faculty respondents recommended 2 and represents a statistically significant difference (p = 0.01).

**Table 3:** Faculty perspectives on the appropriateness, broadness and entrustment level target for EPA regarding posterior spinal decompression and spinal instrumentation for each specialty.

Specialty	EPA	Stage of training	Proportion considering the EPA appropriate for general practice of this specialty Number/total (%)	Proportion considering the EPA not too broad Number/total (%)	Entrustment Level				
					Proportion considering Level E for attainment of competency Number/total (%)	Proportion considering Level D for attainment of competency Number/total (%)	Proportion considering Level C for attainment of competency Number/total (%)	Proportion considering Level B for attainment of competency Number/total (%)	Proportion considering entrustment Level A for attainment of competency Number/total (%)
**Posterior spinal decompression**									
Neurosurgery	Performing posterior cervical or thoracic decompression	SC	36/38 (94.7)	32/38 (84.2)	29/38 (76.3)	8/38 (21.1)	1/38 (2.6)	0/38 (0)	0/38 (0)
Orthopedic surgery	Performing laminectomy/ decompression	C	8/15 (53.3)	14/15 (93.3)	4/15 (26.7)	6/15 (40.0)	3/15 (20.0)	1/15 (6.7)	1/15 (6.7)
P-value from Test of Proportionality Wilcoxon Rank Sum Test			**0.0003** *	0.39	**0.001** *				
Percentage of All Participants (N = 53) **Number/total (%)**			44/53 (83.0)	46/53 (86.8)	33/53 (62.3)	14/53 (26.4)	4/53 (7.5)	1/53 (1.9)	1/53 (1.9)
**Spinal instrumentation**									
Neurosurgery	Performing procedures utilizing spinal instrumentation including posterior subaxial, posterior thoraco-lumbar, occipito-cervical and anterior cervical	SC	26/38 (68.4)	28/38 (73.7)	25/38 (65.8)	11/38 (28.9)	2/38 (5.3)	0/38 (0)	0/38 (0)
Orthopedic surgery	Performing primary posterior instrumented spine fusions	C	5/15 (33.3)	10/15 (66.7)	5/15 (33.3)	5/15 (33.3)	3/15 (20.0)	1/15 (6.7)	1/15 (6.7)
P-value from test of proportionality Wilcoxon Rank Sum test			**0.02** *	0.62	**0.03** *				
Percentage of all participants (N = 53) **Number/total (%)**			31/53 (58.5)	38/53 (71.7)	30/53 (56.6)	16/53 (30.2)	5/53 (9.4)	1/53 (1.9)	1/53 (1.9)

C = Core of Discipline; EPA = Entrustable Professional Activity; N = Number; SC = Senior core.

*Statistically significant.

Based on total responses across both specialties regarding the PSD, most Faculty stated that PSD was appropriate to the general practice of their specialties and not too broad. Sixty-two percent proposed that entrustment level E should be the expected competence for graduating residents **Table 3.**

Nearly all neurosurgery Faculty (94.7%) believed that PSD is appropriate for general neurosurgery practice rather than fellowship subspecialization, while only 53.3% of orthopedic surgery Faculty considered PSD is appropriate for general orthopedic surgery practice (p = 0.0003). Most neurosurgery Faculty (84.2%) and orthopedic surgery Faculty (93.3%) agreed that PSD is not too broad (p = 0.39). Among neurosurgery Faculty (76.3%) expected entrustment level E as a standard competence performance for graduating residents while only 26.7% of orthopedic surgery Faculty felt the same (p = 0.001). The majority of orthopedic surgery Faculty (40%) considered level D as the target for graduating residents for PSD.

Logistic regression revealed that the Faculty’s expectation towards entrustment level E for PSD associated with the agreement of the Faculty that PSD is appropriate for general practice of their specialties with an odds ratio of 8.35 (p = 0.01, 95% CI 1.53–45.67). This relationship was not observed with other variables.

**Fig 1:**
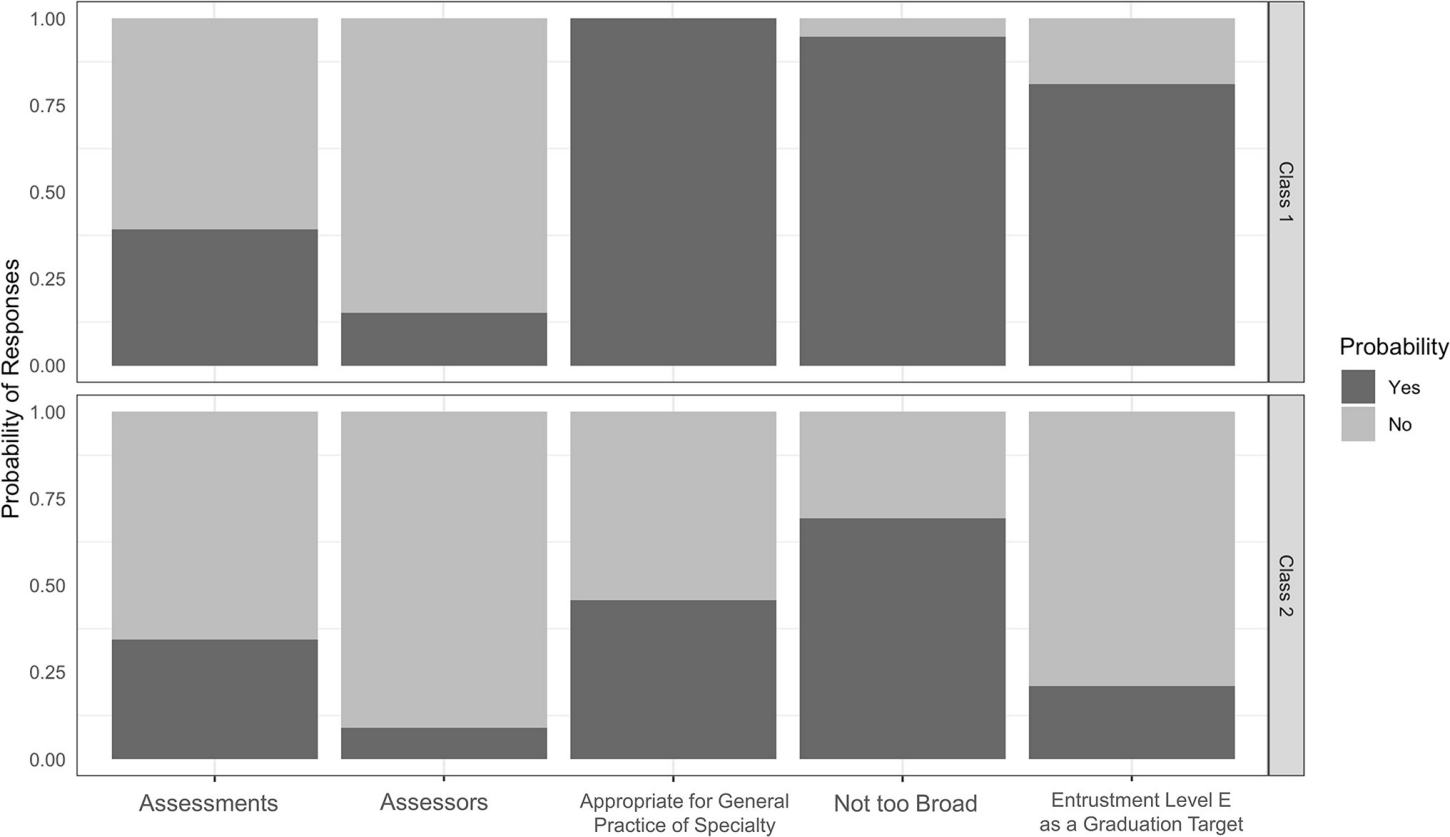
Findings from Latent class analysis for posterior spinal decompression EPA (PSD). EPA = Entrustable Professional Activity; PSD = Posterior spinal decompression Entrustable Professional Activity.

Latent class analysis revealed two classes of respondents **(Fig 1).** Faculty in class 1 tended to believe that PSD was appropriate for general practice of their specialty and consider level E as expected standard competence performance.

Class 1 was comprised of Faculty with a high probability of thinking that PSD was appropriate for general practice and considering level E as a graduation target. In class 2, Faculty had a high probability of thinking that PSD was appropriate for fellowship training and not expecting graduating residents to achieve level E. Both classes demonstrated a high probability of believing that PSD was not too broad, not expecting more assessments and assessors than the average.

**Table 4** shows that neurosurgery Faculty comprise the majority of class 1 and the majority of class 2 is orthopedic Faculty. The Faculty in class 1 are more likely to consider PSD appropriate for general practice of their specialty, not too broad and more expectation toward entrustment level E than the Faculty in class 2.

**Table 4:** Characteristics of Faculty in class 1 and 2 from Latent class analysis for PSD.

	Class 1 (N = 42)	Class 2 (N = 11)	P-value
	N (%)	N (%)	
**Specialty**			
Orthopedic surgery	8 (19.0)	7 (63.6)	**0.003** *
Neurosurgery	34 (81.0)	4 (36.4)	**0.004** *
**Working experience**			
< 5 years	9 (21.4)	2 (18.2)	0.82
5–10 years	5 (11.9)	2 (18.2)	0.60
11–20 years	15 (35.7)	2 (18.2)	0.28
>20 years	13 (31.0)	5 (45.5)	0.38
**# Faculty practicing at participant’s hospital (mean)**	10.6	10.2	0.77
**# Residents on participant’s specialty service at participant’s hospital (mean)**	5.5	5.5	0.89
**# Residents supervised by participant per day (mean)**	1.7	1.6	0.92
# Faculty believed that the current EPA was appropriate for training	42 (100)	2 (18.2)	**< 0.001** *
# Faculty believed that the current EPA was not too broad	40 (90.9)	6 (54.5)	**< 0.001** *
# Faculty considered Entrustment level E as reflecting attainment of competence	31 (73.8)	2 (18.2)	**< 0.001** *
# Assessments (mean)	6.6	7.1	0.70
# Assessors (mean)	2.7	2.5	0.56

EPA = Entrustable Professional Activity; N = Number; PSD = Posterior spinal decompression Entrustable Professional Activity.

*Statistically significant.

### Spinal instrumentation EPAs (SI)

**Tables 2 and 3** present results regarding **SI.** Neurosurgery Faculty recommended a median number of 5 assessments and their orthopedic counterparts recommended 4 (p = 0.17). The median number of assessors recommended by neurosurgery Faculty was 3, while orthopedic faculty respondents recommended 2 (p = 0.04).

**Based on total responses across both specialties regarding SI,** slightly more than half felt SI is appropriate to the general practice of their specialties and expected level E as standard competence performance for SI. Seventy-one percent considered SI not too broad.

Over two-thirds (68.4%) of neurosurgery Faculty believed SI was appropriate for general neurosurgery practice, while only one-third (33.3%) of orthopedic surgery Faculty held the same opinion for their general practice (p = 0.02). The majority of neurosurgery and orthopedic surgery Faculty considered SI not too broad, 73.7% and 66.7%, respectively (p = 0.62). A greater proportion of neurosurgery Faculty than those from orthopedic surgery believed that level E is the graduation target for SI (65.8% vs 33%; p = 0.03) Equal proportions of orthopedic surgery Faculty judged level E (33.3%) and level D (33.3%) as a graduating target for SI.

Logistic regression showed no association between Faculty’s expectation towards entrustment level E for SI with any variables.

**Fig 2:**
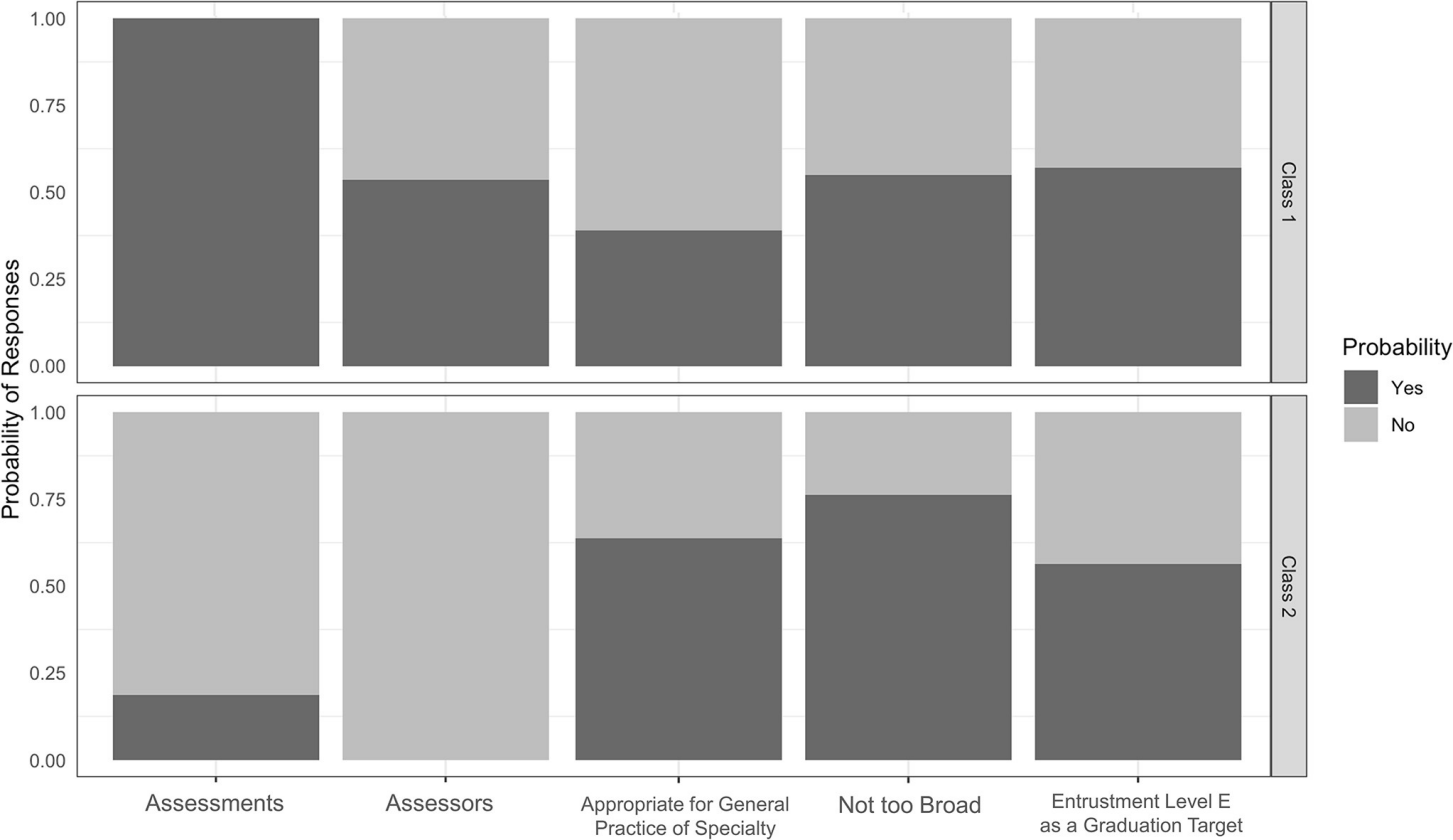
Findings from Latent class analysis for spinal instrumentation EPA (SI). EPA = Entrustable Professional Activity; SI = Spinal Instrumentation Entrustable Professional Activity.

Latent class analysis revealed two classes of Faculty **(Fig 2).** Faculty in class 1 were more likely to believe that SI was rather appropriate for fellowship training than residency training and require more assessments and assessors than the average to demonstrate mastery of SI than Faculty in class 2.

Class 1 was comprised of Faculty who showed a high probability of considering SI more appropriate for fellowship training than residency training, needed more assessments and assessors than the average. In class 2, there was high probability of considering SI appropriate for general practice of their specialty, and not requiring more assessments and assessors than average. Both classes have high probability of thinking SI not too broad and considering level E as a graduation target.

**Table 5** shows that the Faculty in class 1 required more assessments and assessors while the Faculty in Class 2 were likely to consider SI not too broad.

**Table 5:** Characteristics of Faculty in Class 1 and 2 from Latent class analysis for SI.

	Class 1 (N = 9)	Class 2 (N = 44)	P-value
	N (%)	N (%)	
**Specialty**			
Neurosurgery	7 (77.8)	31 (70.5)	0.67
Orthopedic surgery	2 (22.2)	13 (29.5)	0.67
**Working experience**			
< 5 years	2 (22.2)	9 (20.5)	0.92
5–10 years	2 (22.2)	5 (11.4)	0.40
11–20 years	1 (11.1)	16 (36.4)	0.15
>20 years	4 (44.5)	14 (31.8)	0.48
**# Faculty practicing at participant’s hospital (mean)**	11	7.6	0.08
**# Residents on participant’s specialty service at participant’s hospital (mean)**	5.2	5.5	0.91
**# Residents supervised by participant per day (mean)**	1.9	1.7	0.63
# Faculty believed that the current EPA was Appropriate for Training	3 (33.3)	28 (63.6)	0.10
# Faculty believed that the current EPA was not too broad	3 (33.3)	35 (79.5)	**0.005** *
# Faculty considered Entrustment level E as reflecting attainment of competence	4 (44.4)	26 (59.1)	0.43
# Assessments (mean)	16.1	6	**< 0.0001** *
# Assessors (mean)	4.7	2.2	**< 0.0001** *

EPA = Entrustable Professional Activity; N = Number; SI = Spinal Instrumentation Entrustable Professional Activity.

*Statistically significant.

The IQR of the median recommended number of assessments and assessors did not vary between the EPAs (PSD and SI) or the specialties (neurosurgery and orthopedic surgery) **Table 2.** This indicates that there was no significant variation in perspectives of Faculty in each specialty.

## Discussion

We found more uniformity with the number of assessments required of each resident for each EPA than the number of assessors and level of mastery expected of the graduating resident. PSD appears to be an EPA commonly associated with general practice in both neurosurgery and orthopedic surgery and with Faculty expecting relatively high levels of competence (Level E or D) at graduation. In contrast, SI is seen as a more complex procedure and considered differently by the two specialties. Neurosurgeons more commonly consider the SI as one appropriate for general practice with high expectations for competence, whereas fewer orthopedic surgeons see SI as a component of general practice and have lower expectations of mastery for graduating residents.

At the same time, very few, 15.8% of neurosurgery Faculty and only 6.7% of orthopedic Faculty, indicated that PSD was too broad and requires significant revision.

One neurosurgery Faculty stated:

“*This is too broad. This also includes* ***wider postero-lateral thoracic decompression for anterior pathology, which involves resection of the facet joints and pedicle*** *[which] is fellowship level while the first part (patient positioning, level confirmation, removal of lamina while preserving uninvolved ligaments and respecting the spinal cord) is junior resident level.”*

While the RCPSC committee in neurosurgery has specified 8 assessments are required for SI, the orthopedic committee has stipulated that one is sufficient [14,15]. This is reversed when examining the number of assessors; RSPSC requires only two, however the neurosurgery Faculty in this study recommended a minimum of 3 [14]. It could be that the respondents in our survey are self-selected with different characteristics from those who develop policy at the RCPSC. These numbers also differ from the Intercollegiate Surgical Curriculum Programme (ISCP) of the United Kingdom (UK) that requires 10 assessments of complex spinal fusion for graduating neurosurgery residents [16].

Faculty often require clarification about the difference between the “EPA” which defines the task of the specialty, from the “Milestone” which indicates the ability expected at a certain level of training. This distinction between EPA and milestone is further highlighted by the neurosurgery Faculty who suggested the spine-related EPAs should be broken down to several tasks with proper complexity for each level of trainees.

As one respondent remarked:

“*JR [junior resident] should know how to set up the case and open. SR [senior resident] should be able to decompress lamina. CR should know how to remove facet and drill out pedicle under observation*.”

This belief aligns with current neurosurgery EPAs of RCSPC which divide spinal procedures into smaller tasks for different training levels. For example, “performing midline posterior subaxial spinal column exposure and closure” (Foundation), “performing lumbar laminectomy” (Junior Core), “exposing the anterior cervical spine (Junior Core),” as well as orthopedic surgery that has basic spine EPA “performing posterior spinal column exposure and closure” (Core) [14]. In England, the expectation competence performance of spine surgery early orthopedic residents is level 1 (having observed or knowing of) or level 2 (can manage with assistance) for lumbar decompression without fusion or discectomy, while level 4, being able to manage without assistance including potential common complications, is the standard of competence performance for the final-year residents doing a specialty interest in spine [17].

The neurosurgery SI EPA includes more complex fusion (e.g., occipito-cervical fusion). This might contribute to the lower proportion of neurosurgeons who consider SI appropriate for general neurosurgery practice compared with PSD. As one neurosurgery Faculty said:

“*I expect a resident aiming for a general neurosurgery practice to be capable of doing an ACDF 2 levels as well as a 2–3 level PL fusion (pedicle screws/ lateral mass screws). Maybe an OCF as well, All other activities PLIF/XLIF etc.) are better taught at the fellowship level.”*

This possibility is reinforced by the finding that over 90% believe that PSD is appropriate for the general practice of their specialty. However, when compared with orthopedic Faculty, significantly greater proportion of neurosurgery Faculty believe that both PSD and SI are appropriate for general practice of their specialty.

These differences in perspectives may be rooted in the distinct residency training and practice patterns of neurosurgeons and orthopedic surgeons. In Canada, although both types of residencies have five core clinical years of training, the focus of that training is quite different. [24,25]. Neurosurgery residents begin exposure to spine surgery as early as their first postgraduate year (PGY-1) and continue to get a significant exposure to spine surgery in every neurosurgery rotation until they graduate from residency. Since about half of the surgical cases done by practicing neurosurgeons are spinal and half are cranial [26], neurosurgical trainees get a broad and in-depth experience over their five years of training in spinal surgery. In contrast, orthopedic surgery training encompasses a broader range of operations, such as orthopedic trauma, foot and ankle surgery, hip and knee reconstruction, hand and upper extremity surgery, and sports surgery, but with significantly less time dedicated to spine surgery compared to neurosurgery residency [25–29]. Typically, Canadian orthopedic surgery residents spend a total of about three months on spine surgery rotations during their residency, unless they choose to pursue an elective in that area. These facts are supported by a survey of Canadian residents, which revealed that neurosurgery residents spend 37% of their time performing spinal cases, whereas orthopedic residents reported that they devote only 16% of their time to spine procedures [28]. We believe that these differences in training and practice may help to explain the differences that we identified in our study.

The American situation has many parallels to the Canadian training situation in terms of similarities and differences in neurosurgery and orthopedic residency training. A retrospective review of ACGME case logs from 2014–2019 showed that, on average, neurosurgical residents logged 6.8 times the number of spine surgery cases and spent 6.1 times more operating hours on spinal procedures compared to orthopedic residents [29]. These differences in residency training between neurosurgery and orthopedic surgery are also evident in the Intercollegiate Surgical Curriculum Programme (ISCP) in the United Kingdom. The ISCP sets higher requirements for graduating neurosurgery residents, mandating 10 assessments of complex spinal fusions, while orthopedic residents are required to complete only 20 mixed cases of nerve decompression, which can include carpal tunnel, cubital tunnel, tarsal tunnel, spinal decompression, and discectomy [16,17].

Given that neurosurgery residency programs provide more extensive exposure to spine surgery than orthopedic surgery residency programs, graduating neurosurgery residents are likely to feel more confident in performing spine surgeries than orthopedic residents. This perspective is supported by the study’s results, which indicated that Canadian senior neurosurgery residents rated their competence in performing spinal procedures higher than orthopedic residents did, with scores of 79% versus 56% of the maximum possible score (p < 0.001) [28]. Thus, it is not surprising that nearly all neurosurgery Faculty in our study expect that graduating residents should be able to perform PSD and SI independently and they expect residents to perform this at a high level (D or E) by graduation. We posit that given the similarities of training in North American, that our Canadian results would be generalizable to the American situation and other training situations internationally with similar educational programs and practice patterns.

As one orthopedic surgeon who felt that the EPAs should be a component of fellowship training said: *“I don’t think we should expect residents to able to do this completely autonomously*.*”* Another orthopedic surgeon said, *“It is essential for the general orthopaedic surgeon to be able to identify a cauda equina and be able to refer to subspecialty care*.*”*

These points bring to the fore the issue of the role of fellowships and the relative balance of what is mastered in residency versus fellowships. Given the differing perspectives mentioned by neurosurgeons and orthopedic surgeons above, there is little dispute that fellowship is a time to master procedures that are more complex. However, given that neurosurgeons have expertise in cranio-cervical junction, intradural pathology, microsurgery, neurotrauma, and cerebrospinal fluid management while orthopedic surgeons are highly specialized in bone biology, biomechanics, joint arthrosis, instrumentation and spinal deformity, fellowships should be complementary to the training received in residency. Thus, ideally, orthopedic residents would spend more time with neurosurgery Faculty and neurosurgeons more time focusing on tasks with which orthopedic Faculty are recognized experts. Initiatives such as the Area of Focused Competence in Spine Surgery created by the RCPSC holds great hope to move the training of spine surgeons forward [30]. To be successful, it and other similar programs will have to create EPAs and milestones that take into account the strengths that starting fellows come into fellowship with from their residencies and then build complementary experiences to help shape a more well-rounded spinal expert rather than just extend residency programs with more of the same. If competency-based education fosters this educational achievement, then it will also catalyze the hope of moving the whole field of clinical spine management forward. Ultimately, patients should get high quality care regardless of the specialty of their spinal surgeon.

## Limitations

This study was performed in Canada where the curricula and evaluation methods differ from other jurisdictions. Although there are great similarities across nations, different results may have been possible had we surveyed internationally. While we did not ask respondents about subspecialty interests, we specifically sought out opinions of what would be required of the graduating undifferentiated general resident. Our sample size was limited but given the small numbers of specialists in Canadian orthopedics and neurosurgery [31,32], our completion rate was reasonable. Furthermore, we sought out respondents from the academic surgery programs across Canada in both specialties which make up about a half of neurosurgery hospitals and far fewer than half of hospitals where orthopedics is performed. In addition, only a small minority of orthopedic surgeons actually perform spinal surgery, so we believe the sample we received was representative of spinal orthopedics. However, in terms of those teaching residents, which occurs in academic settings, we believe our results are representative. Despite this, we acknowledge that our results could be subject to response bias.

## Conclusion

Difference exists in parameter of assessment and expected standard competence performance of spine procedures among specialties and certifying bodies. The study showed a strong association between Faculty’s perspectives that the selection of entrustment level E as the expected standard of competent performance for graduating residents is strongly related to the idea that PSD is appropriate for general practice of their specialties.

Our results will be particularly valuable to certification bodies in the assessment of spinal milestones. As competency demands by the public mature internationally, our results have an important role in defining standards of competence and the criteria to assess the performance of graduating residents and in so doing, help to standardize quality regardless of the surgeon’s specialty. This study has important implications for the design of residency and fellowship education and evaluation in spinal surgery internationally.
